# The selective dynamics of interruptions at short tandem repeats

**DOI:** 10.1093/genetics/iyag080

**Published:** 2026-03-25

**Authors:** Michael E Goldberg, Harriet Dashnow, Kelley Harris, Aaron R Quinlan

**Affiliations:** Department of Human Genetics, University of Utah, 15 N 2030 E, Salt Lake City, UT 84112, United States; Department of Human Genetics, University of Utah, 15 N 2030 E, Salt Lake City, UT 84112, United States; Department of Biomedical Informatics, University of Colorado Anschutz Medical Campus, 1890 N. Revere Court, Aurora, CO 80045, United States; Department of Genome Sciences, University of Washington, 3720 15th Ave NE, Seattle, WA 98195, United States; Computational Biology Division, Fred Hutchinson Cancer Research Center, 1100 Fairview Ave N, Seattle, WA 98109, United States; Department of Human Genetics, University of Utah, 15 N 2030 E, Salt Lake City, UT 84112, United States

**Keywords:** mutation, evolutionary genomics, repetitive elements

## Abstract

Short tandem repeats (STRs) are hotspots of genomic instability that mutate at rates orders of magnitude greater than nonrepetitive loci due to frequent replication slippage. Expansions at some STR loci cause Mendelian diseases, while variation at other noncoding loci may affect complex traits, possibly by altering transcription factor occupancy of nearby binding sites. Accordingly, some STRs are inferred to be under purifying selection, regardless of their instability. One or more “interruptions”, or bases that disrupt the locus's canonical repeat, significantly decrease an STR's mutability. For example, the onset of Huntington's Disease, a neurodegenerative disorder associated with somatic expansions of a trinucleotide coding STR, is delayed in individuals whose inherited alleles contain interruptions. Thus, interruptions that decrease mutation rate at some coding loci may broadly protect against deleterious phenotypes associated with locus instability. However, interruptions may themselves be deleterious at constrained loci, particularly at noncoding loci in gene regulatory elements, possibly disrupting the formation of secondary structures key to their function. We therefore hypothesized that the frequency of interruptions could depend on a locus's functional importance–at constrained loci, the fitness effects of expansions but also interruptions could be more deleterious than at neutral loci. To test this hypothesis, we examined the distribution of interruptions at ∼650,000 autosomal STRs. In the ∼2,500 3- or 6bp-motif coding STRs, we find that synonymous interruption density increases with purifying selection on the gene, while the opposite is true for missense-causing interruptions. In contrast, noncoding STRs in gene regulatory elements harbor fewer interruptions than elements that are unassociated with gene regulation and thus more likely to be evolving neutrally. Our findings indicate that the abundance of interruptions may be partially explained at coding STRs by the benefit of lower instability, whereas maintaining a core stretch of uninterrupted repeat may be key to the function of regulatory noncoding STRs, outweighing the benefits of stability.

## Introduction

Short tandem repeats (STRs) are repetitive elements that comprise tandem 1–6 bp motifs and account for 3% of the human genome (International Human Genome Sequencing Consortium et al. 2001). In the pregenomic era, STRs were commonly used as population markers and for DNA fingerprinting thanks to their high rates of heterozygosity (Weber and Wong 1993; Spencer et al. 2000). Their high heterozygosity results from remarkable genomic instability; STRs mutate at rates orders of magnitude greater than nonrepetitive loci due to frequent replication slippage, most recently estimated around 5.5 × 10^−6^ mutations/site × generation (Weber and Wong 1993; Sun et al. 2012; Willems et al. 2017; Mitra et al. 2021; Steely et al. 2022; Kristmundsdottir et al. 2023; Porubsky et al. 2025). Loci differ considerably in their mutation rate associated with a number of local features such as GC content, motif length, and allele length (Sun et al. 2012; Ananda et al. 2013; Willems et al. 2017; McGinty and Sunyaev 2023; Porubsky et al. 2025). Due to their repetitive nature, STRs are more likely than high-complexity genomic sequence to form bulky, non-B conformations of DNA, such as hairpins or cruciforms (Trinh 1991; Khristich and Mirkin 2020; McGinty and Sunyaev 2023). These bulky structures likely contribute to replication fork stalling, which results in slippage of the nascent strand. Slippage may result in contraction or expansion of an allele by 1 or more motifs relative to the template strand. Though slippage resulting in mutagenesis is typically linked to cell division, STRs may also expand or contract outside of S-phase replication (Goldberg et al. 2024; Handsaker et al. 2025).

A handful of local characteristics are known to determine an STR's mutation rate. Longer alleles, shorter motifs, and lower GC content are generally associated with higher mutation rate; other characteristics and loci that act in *cis* or *trans* have also been described (Brinkmann et al. 1998; Legendre et al. 2007; Sun et al. 2012; Ananda et al. 2013; Kristmundsdottir et al. 2023; McGinty and Sunyaev 2023). Interruptions, or allelic impurity, refer to substitutions or small insertions or deletions that disrupt an otherwise pure repeat motif and are known to slow down the rate of slippage (Gacy et al. 1995; Sainudiin et al. 2004; Ananda et al. 2014; Wright et al. 2019; McGinty and Sunyaev 2023; Rajan-Babu et al. 2024; Danzi et al. 2025; McGinty et al. 2025). Though the exact mechanism is unclear, interruptions may decrease the likelihood of forming non-B conformations and therefore the expected rate of replication stalling and slippage (Gacy et al. 1995; Pearson et al. 1998). As such, mutation rate roughly scales with the longest remaining stretch of pure repeat (Ananda et al. 2014; Wright et al. 2019). Although single base interruptions may be caused by mutational processes independent of STRs, nucleotides added during STR expansion are erroneous at a rate of 10^−2^ to 10^−3^ per added nucleotide; contractions may also delete interruptions (McGinty and Sunyaev 2023).

Variation at 60 STR loci in coding exons has been long associated with a number of Mendelian diseases typically involving neurodegeneration linked to somatic expansion following inheritance of an allele at or beyond pathogenic length (Hiatt et al. 2025). Huntington's Disease (HD), for example, is linked to somatic expansions at a CAG trinucleotide repeat encoding a polyglutamine stretch in the *HTT* gene (Wright et al. 2019). Individuals who inherit an allele that contains 40 CAG motifs or greater are at risk of disease; the greater the length, the earlier the age of onset due to the higher slippage rate per unit time. However, individuals who inherit an allele with a CAG>CAA interruption have a significantly increased age of onset conditional on the length of the polyglutamine. Similarly, interspersed AGG repeats stabilize *FMR1* and decrease the probability of intergenerational expansion that leads to Fragile × syndrome (Eichler et al. 1995; Eichler and Nelson 1996). Broadly, interruptions stabilize the repeat, protecting it from the slippage that results in deleterious phenotypes (Rajan-Babu et al. 2024; Chen et al. 2024b).

Intronic and intergenic noncoding STRs were historically assumed to be largely nonfunctional, outside of CG-rich repeats at which methylation is associated with gene regulation. However, a number of recent studies have reported that noncoding STR variation has causal links with a number of complex traits such as developmental disorders, cancer risk, and height (Gymrek et al. 2016; Fotsing et al. 2019; Mukamel et al. 2021, 2023; Grasberger et al. 2024). Variation at some STRs directly causes variance in gene expression (Fotsing et al. 2019; Margoliash et al. 2023; Tanudisastro et al. 2025). A proposed mechanism behind these molecular and trait associations is that STRs may alter gene expression when proximal to the binding motif of transcription factors and other DNA binding proteins (Horton et al. 2023). In vitro work has shown that these proteins can directly bind STRs, thus increasing the binding occupancy of a neighboring motif at an enhancer or other *cis*-acting gene regulatory element (Horton et al. 2023). Different proteins bind more strongly and for a longer amount of time to different STR motifs; evolutionary similarity between proteins does not always predict similar motif preferences (Horton et al. 2023). Although purity broadly correlates with stronger binding, the effect is nonmonotonic (Horton et al. 2023). Accordingly, a proportion of these noncoding STRs are inferred to be evolving under purifying selection.

Identifying constraint at STR loci has historically been challenging due to their rapid mutagenesis and high diversity. Many methods identify constrained high-complexity genomic regions as those depleted for SNP variation under a neutral model of their mutagenesis (Lek et al. 2016; Havrilla et al. 2019; Karczewski et al. 2020; Chen et al. 2024a). LOEUF, for example, quantifies constraint at transcripts and genes by estimating the depletion of observed loss-of-function alleles relative to neutral expectation (Karczewski et al. 2020). This score represents a locus's intolerance to dominant loss-of-function. New models that directly identify STR constraint similarly rely on models of their complex mutagenesis and require making similar assumptions of dominance (Gymrek et al. 2017; Mitra et al. 2021; Haasl and Payseur 2024).

**Fig. 1.**
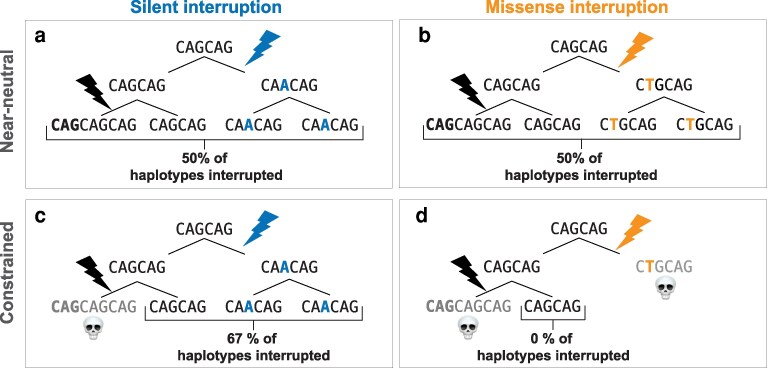
The hypothetical outcome of an interrupted STR allele depends on locus constraint and the functional effect of sequence impurity. Imagine 2 coding STRs with identical mutational dynamics composed of a trinucleotide CAG repeat, encoding a polyglutamine. One locus (a, b) is evolving neutrally or nearly neutrally while the other (c, d) is evolving under selective constraint. G → A single-nucleotide substitution results in an allele at both loci with a silent interruption, which stabilizes the allele and lowers the rate of expansion or contraction (a, c). While all alleles are equally fit at the locus evolving nearly neutrally, expanded alleles are deleterious at the constrained locus and are lost. After several rounds of transmission, the silent interruption is more common at the constrained locus than at the nearly neutral locus. However, a second scenario (b, d) could be considered if an allele arises with a missense interruption, such as an A → T substitution. Again, all alleles are tolerated at the nearly neutral locus, resulting in 50% of alleles carrying an interruption. While an interruption lowers the probability of a deleterious expanded allele at the constrained locus, the missense change is itself deleterious; the allele carrying this interruption is therefore lost through purifying selection. Thus, missense interruptions are less common at the constrained locus than at the nearly neutral one.

The selective dynamics of a genetic element that controls mutation rate were initially derived by Kimura in 1967 (Kimura 1967). A “mutator” is a genetic element that controls the mutation rate of a genomic region or set of elements; excluding pleiotropy beyond its effect on germline mutation rate, a mutator allele's selection coefficient scales with the change in mutation rate, the distribution of fitness effects of those mutations, and the linkage to the mutations. Interruptions stabilize an STR allele in a way that mirrors a *cis*-acting “anti-mutator” in perfect linkage to the mutations whose rate it decreases. If slippage at a locus is deleterious, interruptions that stabilize an allele may therefore be under indirect positive selection (Fig. 1). However, interruptions may themselves be deleterious and under direct purifying selection at constrained loci, particularly at noncoding loci in gene regulatory elements where an interruption would perturb the purity that may be key to their function. We would hypothesize, therefore, that the density of interruptions at STR loci could scale with constraint. At loci evolving entirely or nearly neutrally, expanded and contracted alleles and pure and interrupted alleles are as fit as an unmutated allele, so interruptions that increase stability and decrease purity could fix at a rate only affected by drift (Fig. 1a and b). However, at loci evolving under constraint, we could see either of 2 effects: if expanded and contracted alleles are more deleterious than an interrupted allele of the ancestral length, constrained loci could be enriched for interruptions due to a higher probability of fixation than expected under neutrality (Fig. 1c). The opposite could occur if impurity is more deleterious than expanded and contracted alleles (Fig. 1d). Furthermore, this effect could scale monotonically with constraint and be strongest at loci where purifying selection is highest and intermediate where constraint is mild but nonzero.

To test this hypothesis, we examined the distribution of interruptions at the major alleles of ∼650,000 human autosomal STRs in large population databases. In the ∼2,500 STRs that fall within coding exons and comprise 3- or 6-bp motifs whose expansions and contractions would not change reading frame, we find that the density of interruptions in coding STRs increases with purifying selection on the gene, provided those interruptions lead to no amino acid change. In contrast, noncoding STRs under purifying selection harbor fewer interruptions than those evolving neutrally. Our findings indicate that the abundance of interruptions may be partially explained at coding STRs by the benefit of a lower mutational burden at their linked loci. On the other hand, maintaining a minimum core stretch of uninterrupted repeat may be key to the function of noncoding STRs that fall within regulatory elements, outweighing the benefits of lowering the mutation rate.

## Methods

For the majority of analyses, we relied on STR genotypes generated by EnsembleTR on 3,550 diverse genomes from the 1000 Genomes (1KGP) and H3Africa (H3A) cohorts (The 1000 Genomes Project Consortium 2015 ; Choudhury et al. 2020; Byrska-Bishop et al. 2022; Jam et al. 2023). Both cohorts contain short-read WGS aligned to GRCh38; the 1KGP samples are PCR-free. EnsembleTR integrates the output of 4 different TR genotyping methods; of these 4, HipSTR is the only tool that infers and reports the exact sequence of an allele rather than estimating its length alone; this resolution is necessary to detect interruptions (Willems et al. 2017). Thus, we subsetted the loci to those that contained HipSTR genotypes, which are limited in length to those spanned by one end of a paired-end short read (Willems et al. 2017). We excluded related individuals from the 1KGP (https://ftp.1000genomes.ebi.ac.uk/vol1/ftp/release/20130502/). We used similar filters to those applied in Jam et al. (2023) and excluded loci missing genotypes for >25% of individuals, failed Hardy-Weinberg Equilibrium testing (*P* < 0.000001), or overlapped a known segmental duplication. Individual genotypes that did not pass quality filters were also excluded. We exclusively analyzed the major allele at each locus; allele frequencies were recalculated after excluding these individuals. Homopolymer loci were excluded.

### Coding STRs

Data processing differed slightly for the coding and the noncoding STR analyses. For an STR locus to be considered “coding”, the locus needed to be fully contained within an exon from a canonical transcript, a medically-relevant exon, or an exon inferred to be under significant purifying selection; these were aggregated from RefSeq Select and MANE (Morales et al. 2022; Goldfarb et al. 2025). To calculate the number of interruptions, we calculated the number of interrupted codons relative to a pure allele, as described in the main text (Supplementary Fig. 1). Codons differ in their “mutational space”, i.e. the fraction of possible substitutions that lead to a silent vs a missense mutation. We estimated this fraction by counting every possible substitution from the pure sequence and binned by coding consequence, excluding nonsense mutations. Major alleles at which over 25% of bases were classified as interruptions were excluded.

We used several different annotations to approximate constraint at coding STRs. Predominantly we relied on the containing gene's LOEUF score, which is a quantitative measure of a gene's intolerance to dominant loss-of-function mutations (Karczewski et al. 2020). Lower LOEUF scores (LOEUF ∼ = 0) indicate high constraint and a depletion of observed loss-of-function mutations. We assumed here that the selective constraint on the STR would scale with that of its containing gene, as many STR expansion diseases display dominant inheritance patterns (Hiatt et al. 2025). We also intersected STRs with inferred protein domains from Pfam and downloaded the track from the UCSC Table browser (Blum et al. 2025). We used a list of genes at which deleterious alleles behave in an autosomal dominant manner generated previously (Berg et al. 2013).

### Noncoding STRs

To determine purity at noncoding STRs, we calculated the minimum Levenshtein distance between an observed allele and a pure allele, allowing for local alignment to the pure allele to account for insertions or deletions of bases other than a pure motif. All coding STRs were excluded from these analyses. As above, we examined major alleles only and excluded loci whose major allele was over 25% interrupted.

The noncoding STR analyses involved intersecting loci with a variety of annotations, as described below:

1. chromHMM: We downloaded the GRCh38 track for 25 inferred chromHMM states based on observed and imputed experiments on the WA-07 (E002) ESC cell line from https://egg2.wustl.edu/roadmap/web_portal/ (Ernst and Kellis 2012, 2017). State annotations were downloaded from the same website. Some STR loci intersected multiple different states; all loci were classified into a single state using the following sequential logic. Loci that overlapped chromHMM positions with states 1 or 5–9 were classified as transcribed; states 2–4 were promoters; 13–15 were strong enhancers; 10–12 and 16–18 were weak enhancers; 19, 20, 22, or 23 were “other”, and the remaining loci were classified as heterochromatin.
2. DNA binding protein CHiP-seq: we downloaded conservative IDR thresholded peaks from ENCODE for 10 different DNA binding proteins assayed in HeLa cells, the cell type used in Horton et al. (2023). Accession numbers and filenames for each experiment are in Supplementary Table 1.
3. iPSC ATAC peaks: we downloaded ATAC footprints in WTC11 iPSC cells from ENCODE (accession ENCSR506RMU).
4. FIRE peaks: we downloaded GM12878 and K562 FIRE peaks from https://stergachislab.github.io/Fiber-seq-publication-data/ (Vollger et al. 2025).

### Non-B DNA structure prediction

nBMST is a computational tool that predicts whether a certain non-B DNA structure could form at a locus given its nucleotide sequence (Cer et al. 2011, 2012). The program processes a nucleotide sequence and reports the location(s) within that match a pattern required to form one of a variety of non-B structures. For each nonhomopolymer STR locus, we extracted the sequence of the major allele in the 1KGP & H3A, as in the majority of our models. We surrounded each allele by a margin of 150 bp of GRCh38 reference on either side to capture non-B structures forming over a pattern including both repetitive and nonrepetitive sequence. For each allele, we ran nBMST to predict 6 possible shapes: slipped strand (direct repeats), triple helix (mirrored repeats), cruciform (inverted repeat), bent DNA (A-phased repeats), Z-DNA, and G-quadruplex. For each structure, alleles were binned by whether ≥1 bp of repetitive sequence was predicted to form a structure. We ignored non-B structures predicted to form exclusively in the margin on either side of an STR allele that did not extend into the repetitive allele.

We note that the regression models used throughout this study already correct for nucleotide content, allele length, and repeat unit length, therefore sharing some of the parameters used by nBMST to predict shape. However, the covariates in our regression models typically capture linear or monotonic effects; the probability of specific shape formation is not always so simple. Z-DNA, for example, forms at purine-pyrimidine sequences. This pattern exists exclusively at STR alleles containing motifs of an even length; modeling repeat unit length as a monotonic covariate would not capture this pattern.

### SISTR analysis

We further analyzed the effects of noncoding STR constraint on interruptions using a set of orthogonal methods to those described above. We used SISTR scores as a measure of STR constraint (Mitra et al. 2021). SISTR infers the selective constraint at a locus by detecting lower diversity than expected given a model of mutations (expansions and contraction) under neutrality. For each locus, the score *s* refers to the expected decrease in selection coefficient associated with a difference in length of one repeat from the major allele. Scores are estimated using approximate Bayesian computation on forward-time simulations under these models of STR diversity (Mitra et al. 2021). SISTR scores are reported exclusively for 2-, 3-, and 4-mer STRs; of these, 62,729 loci had scores with a 95% confidence interval (CI) of <0.3; higher CIs indicate a noisy estimate. Loci were classified as evolving neutrally or under constraint if their median *s* was 0 or > 0, respectively. Loci that overlapped coding regions were excluded.

To calculate purity, we used segregating SNVs identified in gnomAD v3 that overlapped the reference allele of the above noncoding loci (Chen et al. 2024b). SNVs that did not pass quality filters or were singletons were excluded.

### Regression framework

All statistical modeling was completed in R version 4.4.2. Unless otherwise noted, regressions were Poisson GLMs with a log link function and included motif GC content and length (in bp) as covariates and allele length as an offset variable.

We used a slightly more complex model to account for codon-specific differences in mutational space while analyzing the effects of constraint on coding STRs. For any given codon, the probability that a given single base substitution would result in a silent mutation is lower than that of a missense mutation. Therefore, identifying more missense than silent interruptions could be biased by this mutational space. The number of missense interruptions for a coding allele was reweighted by the ratio of possible silent to missense single base substitutions. In Poisson GLMs that jointly modeled the distribution of both silent and missense interruptions, the offset variable for missense interruptions in a given allele was the product of that ratio and the number of codons.

### Code availability

All code to run these analyses and generate manuscript figures is available at https://github.com/goldmich/str_interruptions.

## Results

### Coding STRs in constrained genes are enriched for silent interruptions

Interruptions that stabilize an STR allele at a small number of disease-related loci are protective against deleterious phenotypes associated with their slippage (Eichler et al. 1995; Wright et al. 2019 ; Chen et al. 2024b). We hypothesized that this phenomenon may generalize throughout a genome and that interruptions would be favored at coding loci under constraint, as long as they did not change the protein coding sequence (Fig. 1). Interruptions that cause missense mutations to a protein coding sequence could be less tolerated, as the selective benefit of a more stable locus may be outweighed by the selective cost of changing an amino acid. However, at loci evolving neutrally or nearly neutrally whose alleles are equally fit regardless of their length, we hypothesized that interruptions would not be selectively beneficial and accumulate under a neutral model of mutagenesis (Fig. 1a and b). If this hypothesis is correct, we would observe that locus constraint covaries with its density of interruptions and their protein coding consequence.

To test this hypothesis, we examined 2,838 trinucleotide and hexanucleotide STR loci whose allele in the GRCh38 reference genome was fully contained in an exon from a canonical transcript in RefSeq Select or in a medically-relevant or constrained exon in Matched Annotation from the NCBI and EMBL-EBI (MANE) (Morales et al. 2022; Goldfarb et al. 2025). STR genotypes from 2,852 individuals of diverse ancestry, comprising all unrelated individuals from the 1,000 Genomes (1KGP) and the H3Africa (H3A) cohorts, were previously generated using HipSTR (Jam et al. 2023). These STRs are short enough to be fully spanned by one end of an Illumina short read and thus represent a certain subset. We filtered loci that were missing genotypes at >25% of individuals, failed Hardy-Weinberg Equilibrium (*P* < 0.000001), or overlapped a known segmental duplication and applied sample-level filters as described in (Jam et al. 2023) and identified the major allele amongst those remaining. Coding major alleles spanned total lengths of 14–104 bp. Limiting this analysis to major alleles genotyped across hundreds to thousands of individuals helped ensure genotyping accuracy which could be compromised at lower frequency alleles.

To count the number of interruptions for each major allele, we took an evolutionary approach assuming that, at some point in time before the observed major allele was derived, this “ancestral” version of the major allele was pure and that interruptions accumulated over time on derived alleles that fix (Supplementary Fig. 1). This method likely overestimates the number of interruptions, but we assume that the degree of overestimation is independent of locus constraint so will not affect our models. We inferred a hypothetical pure allele composed of the locus's motif as detected by HipSTR and of identical length to the observed major allele that minimized the number of amino acid changes between the 2 and excluded nonsense mutations over the theoretical evolutionary history (Methods). For each codon, we compared the nucleotide sequence between the hypothetical pure ancestral allele and determined whether there were any substitutions (i.e. was the codon interrupted), and whether the substitution(s) resulted in a synonymous or missense change. Codons differ in their synonymous “mutational space”; i.e. of all 9 possible individual single-nucleotide substitutions, a different number of them lead to silent mutations. To account for these differences in mutational space, we calculated the ratio of all possible synonymous to nonsynonymous single base substitutions from the ancestral allele. This reweighting strategy corrects for the neutral mutational process rather than accounting for differences in fitness effects, the distributions of which we certainly expect to differ between silent and missense interruptions. For example, an STR allele that contains 1 silent and 2 missense interruptions may appear enriched for missense interruptions. However, if any substitution is 3 times more likely to cause a missense interruption than a silent interruption, the allele actually contains fewer missense interruptions than expected under neutrality. The reweighting strategy simply accounts for this difference in the mutational process. Alleles composed of 25% interrupted bases were excluded. We used the containing gene's LOEUF score as a proxy for locus constraint; LOEUF represents a gene's intolerance to dominant loss-of-function mutation. Low LOEUF values correspond to strong purifying selection, signaled by a depletion of segregating deleterious variants.

**Fig. 2.**
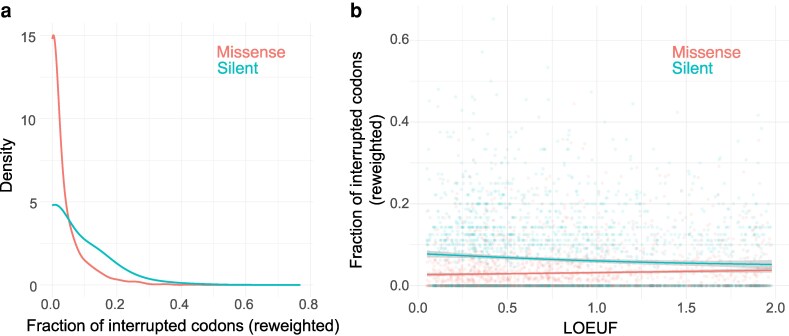
Interruptions at coding STRs. a) Silent interruptions are common and missense interruptions are rare at constrained coding STRs. The numbers of missense and silent interruptions are reweighted based on mutational space. b) Silent interruptions are enriched at constrained genes while missense interruptions are depleted. The fractions of interrupted codons are reweighted by mutational space of silent and missense interruptions, respectively. Fraction of interrupted codons for the major allele at each coding STR is plotted as a function of the containing gene's LOEUF score. Lower LOEUF scores correspond to genes less tolerant of dominant loss-of-function alleles, thus indicating stronger purifying selection.

Of the 2,554 STR loci that fall within genes and are annotated with a LOEUF score, the majority of major alleles are interrupted relative to a hypothetical pure allele (66.1%). LOUEF scores of the containing genes range from 0.051 to 1.978. To determine the effects of constraint on interruptions, we built a series of Poisson generalized linear models (GLMs) with log link functions. These models included 2 covariates to control for characteristics that are known to affect locus stability: motif length and GC content. We controlled for allele length and mutational space as an offset variable. Unless otherwise specified, all GLMs in this manuscript correct for motif length and GC content as covariates. In this section, mutational space and allele length are included in an offset variable. After correcting for these characteristics, we observed that major alleles are enriched for silent over missense interruptions (Poisson GLM with log link, *P* < 2.2 × 10^−16^; Fig. 2a; see Methods).

The number and consequence of interruptions covary significantly with genic constraint. STRs within highly constrained genes have significantly more silent interruptions than STRs in genes evolving more neutrally (Poisson GLM, *B* = −0.24, *P* = 5.34 × 10^−8^). In contrast, constraint nominally decreases the number of missense-causing interruptions (Poisson GLM, *B* = 0.065, *P*=0.070). A GLM with an interaction term finds that silent interruptions are significantly more prevalent than missense interruptions and that constraint has a significantly different effect on the number of interruptions conditional on their coding consequence (Poisson GLM; *B =* 1.39, −0.40; *P* < 2 × 10^−16^, *P* = 3.38 × 10^−12^, respectively; Fig. 2b). Modeling constraint significantly improves a Poisson GLM, indicating that constraint helps explain coding STR purity (ΔAIC = −45).

We examined how alternate measures of coding STR constraint covaried with interruptions and found heterogeneous effects. To independently approximate constraint, we examined whether falling in a gene known to underlie an autosomal dominant (AD) disease affected interruption prevalence (Berg et al. 2013). The 159 loci that fall in AD disease genes are not significantly differently interrupted than the 2,644 loci that do not fall in these genes, regardless of the protein coding consequence of the interruption (Poisson GLM, *P* = 0.50). Relatively few loci fell in AD disease genes, potentially limiting our power to detect any effect on allele purity. We next tested whether an STR locus overlapping an annotated protein domain affected its purity. Given our previous findings described in Fig. 1, we expected to see that STRs in protein domains contained more silent and fewer missense interruptions than STRs outside of annotated domains. Contrary to these expectations, however, the 317 STR loci that fall within known protein domains have significantly fewer silent interruptions (Poisson GLM, *P* = 1.56 × 10^−7^). Perhaps at these highly constrained regions a silent interruption's negative effect on RNA secondary structures and regulation or on tRNA optimization begins to outweigh the benefits of lower mutation rate (Lawrie et al. 2013). The effect of genic constraint on silent interruptions does not significantly differ between STRs within and outside of protein domains (Poisson GLM, *P* = 0.27).

Broadly, we found evidence that selective constraint affects purity at trimer and hexamer STRs contained in genes. STRs in genes under strong constraint are significantly more likely to harbor interruptions, provided that those interruptions arise from mutations that did not change the protein coding sequence. On the other hand, missense-causing interruptions were less common at constrained genic STRs. This interaction supports a hypothesis that interruptions may be selectively beneficial at highly constrained loci by decreasing the local rate of deleterious expansion and contraction through slippage. However, the direct purifying selection of a missense variant may outweigh the indirect positive selection granted by slowing slippage rates, leading to significantly different associations between interruptions and constraint while considering the protein coding consequence.

### Constraint and functional heterogeneously affect purity at noncoding STRs

We then asked if purity was similarly affected by constraint at noncoding STRs. Most noncoding STRs are likely evolving neutrally or nearly neutrally in humans; however, a subset is hypothesized to be under constraint, likely due to their capacity to affect gene regulation (Gymrek et al. 2017; Mitra et al. 2021; Horton et al. 2023). STRs can modify the affinity of DNA binding proteins when they flank the protein's binding motif; the neighboring STR is directly bound by the protein before binding to the motif, thus increasing the amount of time the motif is bound. STRs are further known to be enriched in enhancers (Sawaya et al. 2013; Gymrek et al. 2016; Horton et al. 2023). Expansion and contraction appear to be deleterious at these noncoding loci, so we hypothesized that slowing the causal slippage by decreasing locus purity may be selectively beneficial, as for coding STRs (Mitra et al. 2021). If purifying selection on expanded or contracted alleles is strong, we could expect to see an enrichment for interruptions at constrained noncoding STRs. However, interrupting an STR that flanks a binding motif can heterogeneously decrease the motif's binding occupancy (Horton et al. 2023). Thus, the direct functional consequence of a substitution could possibly outweigh the benefits of slowing slippage. If purity is more selectively beneficial than instability is deleterious, constrained noncoding STRs could be depleted for interruptions.

Similar to the analyses above, we considered the purity of the major allele only at 644,008 nonhomopolymer noncoding STRs. Interruptions were quantified slightly differently than at coding repeats: unable to consider the consequence of each interruption on an amino acid sequence, we calculated the minimum Levenshtein distance to a hypothetical pure ancestral version of the repeat composed entirely of the reference repeat unit. We used a modified alignment approach to account for indels in inferring an ancestral pure allele (Methods). 61.3% of loci harbored at least 1 interruption (Supplementary Fig. 2). As before, we excluded loci whose major alleles were more than 25% interrupted (Levenshtein distance/allele length in bp) as these alleles could be incorrectly genotyped or include multiple major motifs; this roughly corresponded to the 99th percentile of impurity.

Both STR constraint and the functional consequence of an interruption are trickier to quantify at noncoding loci, so we relied on several different annotations. We first examined interruptions across the noncoding genome as a function of their chromatin structure and expected function in gene regulation as identified by chromHMM. To best approximate the chromatin state in the germline, we used 25-state chromHMM annotations inferred from ChIP-seq experiments completed in and imputed for the WA-07 female embryonic stem cell line (Ernst and Kellis 2012, 2017). Roughly 500,000 loci fell in annotated heterochromatin regions; 22,065 and 2,522 fell in weak and strong enhancers, respectively, and 2,748 fell in promoters; the rest of the loci fell in other predicted chromatin states.

**Fig. 3.**
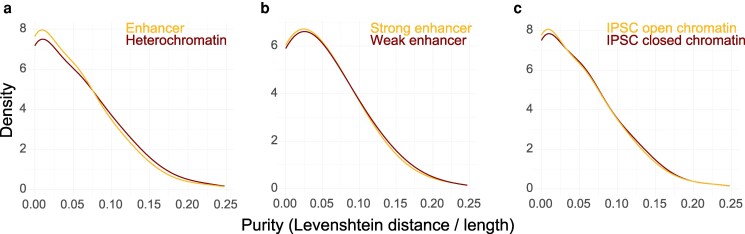
Distribution of interruptions at noncoding STRs stratified by chromatin state and regulatory function. a) STRs that intersect enhancers are purer than those intersecting heterochromatin. b) Inferred enhancer strength in ESCs is predictive of STR purity. c) Open chromatin in iPSCs, approximated by ATAC-seq peaks, predicts significantly purer enhancer STRs.

We hypothesized that STRs in gene regulatory elements may have significantly different purity than those falling in heterochromatin regions; we assume that the latter STRs are evolving more neutrally than the former given their inferred lack of accessibility in the germline. To test this hypothesis, we first modeled the Levenshtein distance of the major allele at an STR locus as a function of whether it coincides with an enhancer or heterochromatic region, after accounting for covariance in length, repeat unit size, and GC content. STRs within enhancers have significantly fewer interruptions than those within heterochromatic regions; furthermore, STRs are significantly purer if they lie in a strong rather than a weak enhancer (Poisson GLMs, *P* < 2 × 10^−16^, *P* = 1.46 × 10^−3^, respectively; *B* = −0.0514, −0.055, respectively) (Fig. 3a and b). STRs in enhancers are also more likely to be completely pure than those falling in heterochromatin (logistic regression, *P* < 2 × 10^−16^; *B* = 0.118). We then segregated the STRs in enhancers by whether they coincide with open chromatin identified by ATAC seq in iPSCs as a proxy for germline activity. STRs in enhancers that also intersected ATAC seq peaks were significantly purer than those in enhancers that did not intersect the peaks (*P* = 0.0207; *B* = −0.0274) (Fig. 3c). However, we did not observe the same effect of enhancer STRs intersecting Fiber-seq Inferred Regulatory Elements (FIRE) peaks called in both GM12878 and K562 cells (*P* = 0.738) (Vollger et al. 2025). The purifying selection placed on STRs by their possible function in gene regulatory elements may be specific to those active in the germline and therefore more identifiable using annotations from iPSC proxies. Interestingly, STRs that intersect promoters have even fewer interruptions than those that intersect enhancers (Poisson GLM, *P* = 0.0214, *B* = −0.038) (Supplementary Fig. 3). Background selection present in promoters would certainly decrease allelic variance at STRs relative to enhancers but could not easily explain differences in purity. This effect on purity at promoters could indicate that the STRs they contain are more likely to be involved in DNA binding protein recruitment than those in enhancers, or that the strength of purifying selection is stronger.

STR mutation rate scales with the longest stretch of pure repeat; interruptions in the middle of a repeat will decrease the probability of slippage more than an interruption closer to the flanks (Wright et al. 2019, 2020). If interrupting an STR in a gene regulatory element incurs a roughly constant cost to protein-DNA binding activity, therefore incurring a set selective cost, we theorized that interruptions would need to maximally lower deleterious slippage to outweigh that cost. Thus, we hypothesized that, conditioning on allele purity, STRs in gene regulatory elements would maintain a shorter pure stretch of repeat. To test this hypothesis, we modeled the longest stretch of pure repeat (in bp) as a function of chromatin state while accounting for covariance with repeat unit length and GC content; as above, we used a Poisson GLM with a log link function with the log-transformed total length of the STR as an offset variable. STRs maintained a significantly longer stretch of pure repeat when intersecting an enhancer relative to intersecting heterochromatin (*P* < 2 × 10^−16^, *B* = 0.014). However, when we conditioned on allele purity by including Levenshtein distance as an independent variable, we found that STRs in heterochromatin maintained a longer pure repeat (*P* = 8.09 × 10^−13^; *B* = 0.0119). AIC values indicated that the model accounting for Levenshtein distance significantly fit our data better than the model without (Δ AIC = 1,034,744).

STR motifs can modify their effect on DNA binding protein occupancy at neighboring binding motifs (Horton et al. 2023). Certain motifs promote significantly stronger and longer binding while others are antagonistic. In vitro experiments have mapped motif preferences for the human DNA binding protein MAX; this mapping is unknown for most other DNA binding proteins (Horton et al. 2023). AG dinucleotide repeats preferentially bind MAX while AC disfavors binding. Given our results above on purity in STRs within enhancers, we hypothesized that, with dinucleotide repeats proximal to a MAX binding site, AG STRs would be less interrupted than AC STRs. Neighboring AG STRs may be more functionally relevant to the occupancy of a MAX binding site and thus under more purifying selection. However, we observe the opposite: AG dinucleotide STRs are significantly more interrupted than AC STRs, conditional on those STRs falling in chromHMM enhancers (Poisson GLM, *P* = 0.0034, *B* = 0.547). We expanded this analysis to include 9 other DNA binding proteins, including MYC, which shares a similar basic helix-loop-helix structure with MAX. Though we do not know which specific motifs promote binding for each of these proteins, we hypothesized that AC and AG dinucleotides would at minimum contrast in their effects modifying binding. After Bonferroni correction for multiple testing, interruptions did not significantly vary as a function of nucleotide content for STRs proximal to any non-MAX binding site. STRs proximal to ELK binding sites were the closest to significance; as above, AG dinucleotide STRs were significantly more interrupted (Poisson GLM; *B* = 0.9178, Bonferroni *P* = 0.096) (Supplementary Fig. 4). AG dinucleotides are broadly more interrupted than AC dinucleotides at STRs near binding sites of a diverse set of DNA binding proteins; this appears to broadly reflect a higher density of interruptions at AG dinucleotide repeats in chromHMM enhancers (Poisson GLM; *B* = 0.2236, *P* < 2 × 10^−16^). We corrected for this bias by introducing an interaction variable to the Poisson GLMs, testing for differences in interruption densities unique to each set of binding sites. After Bonferroni correction for multiple testing, we found that AG STRs proximal to CTCF binding sites had significantly fewer interruptions than expected relative to other enhancers (Poisson GLM with an interaction variable between motif type and proximity to a CTCF binding site, *B* = −0.351, Bonferroni *P* = 0.0311).

Although local DNA shape parameters did not directly explain the propensity of DNA binding proteins to bind to STRs, the probability of forming specific non-B DNA structures could still modify or explain part of the effect of gene regulatory function on purity (Horton et al. 2023). Naturally these bulky non-B DNA structures are fragile and can disrupt replication, thus leading to a higher probability of expansion and contraction (Trinh 1991; Khristich and Mirkin 2020; McGinty and Sunyaev 2023 ). Thus, one might expect that alleles likely to form non-B structures would harbor a higher number of interruptions due to this neutral mutational process. However, formation of these structures could themselves be required for the function of a noncoding STR and therefore be key to the relationship between selection and purity (Prorok et al. 2019; Fleming and Burrows 2020; Maekawa et al. 2022; Makova and Weissensteiner 2023 ). Therefore, we tested how the probability of non-B DNA structure formation could modify the relationship we observed between STRs proximal to enhancers and promoters and interruption density. To predict non-B DNA structure formation in silico genome-wide, we used nBMST to infer the probability of 1 of 6 different shapes (slipped strand, triple helix, cruciforms, bent DNA, G-quadruplex, and Z-DNA) forming at a major allele at each nonhomopolymer STR locus (see Methods) (Cer et al. 2011, 2012). Because nBMST predicts these structures by matching patterns in a nucleotide sequence, the control variables that account for covariance with allele length, GC content, and motif length in our regression models already capture part of the variance of non-B structure formation between loci. Furthermore, in silico tools such as nBMST can have low sensitivity and specificity, inherently failing to capture cell type variance or transient formation of these non-B DNA structures (Li et al. 2009; Cer et al. 2012; Makova and Weissensteiner 2023). Nevertheless, these predicted annotations may still indicate broader patterns in the relationship between non-B structure formation, noncoding STR function, and purity.

We first examined how non-B DNA structures could modify the density of interruptions genome-wide; to avoid possible confounders of strong selection on coding regions, we limited this initial analysis to the noncoding genome. Unsurprisingly, the frequency of non-B DNA structures predicted to form at STR loci varied by shape (Supplementary Fig. 5). Nevertheless, we found that the major alleles at noncoding STR loci were more likely to be interrupted if predicted to form a non-B DNA structure, even after accounting for variance in allele length, GC content, and repeat unit length (Poisson GLM; all Bonferroni *P* < 1 × 10^−14^, *B* = 0.71, 0.84, 1.08, 0.49, 1.24, 0.22, respectively, for slipped strand, triple helix, cruciforms, bent DNA, G-quadruplex, and Z-DNA). These results are generally consistent with the observation that non-B structure formation leads to a higher probability of expansion or contraction, which itself can cause interruptions.

We then modeled how non-B DNA structure formation could better predict or modify the effects of selection and function on purity. To do so, we added an interaction variable to 12 different models, testing each whether STR loci proximal to strong enhancers or promoters that formed 1 of the 6 non-B DNA structures harbored significantly different purity than those that were not predicted to form a shape, respectively. All models except 1 (effect of cruciform formation and proximity to strong enhancers) showed that STRs proximal to promoters or strong enhancers remained significantly purer than those in heterochromatin, regardless of shape formation (Bonferroni-corrected *P* < 5 × 10^−2^; *P* = 0.13 for cruciforms and strong enhancers). Broadly, non-B DNA structures do not significantly modify purity at STRs within strong enhancers. The exception is cruciform-forming repeats, which appear significantly purer when proximal to strong enhancers than repeats that do not form those structures (*P* = 1.16 × 10^−7^). Although this model is technically a better fit to the observed data (Δ AIC = −35,724), the control covariates have opposite signs to our expectations (i.e. repeats of longer repeat units and higher GC fraction have more interruptions) so the results should be treated with skepticism. In contrast, we find that formation of 5 of the 6 non-B DNA structures do significantly modify the effects of proximity to promoters and allele purity. Alleles predicted to form G-quadruplexes or cruciforms that are proximal to promoters are significantly purer than those that do not form the structure; the opposite effect is observed for slipped strand, triple helix, or Z-DNA formation (Bonferroni *P* < 5 × 10^−2^; *B* = −0.26, −0.15, 0.188, 0.144, 0.125, respectively). As before, the covariates have inconsistent signs for the model examining cruciform formation. That G-quadruplex formation is associated with even higher purity at STRs proximal to promoters could indicate that maintenance of their formation is both important to the function of the gene regulatory elements and easily disrupted by interruptions either within or near the structure itself. On the other hand, the association of slipped strand, triple helix, and Z-DNA formation with more interruptions is more puzzling. Because the effect is observed in promoters and not enhancers, perhaps the greater number of interruptions is due to a higher transcription rate relative to STRs containing those structures but in untranscribed heterochromatin. Alternatively, interruptions could be less destabilizing or expansions and contractions are more deleterious to promoters associated with these structures.

We next examined interruptions at enhancers identified by GeneHancer. GeneHancer integrates annotated enhancers from ENCODE, Ensembl, FANTOM, and VISTA and further infers genes whose expression they modify (Fishilevich et al. 2017). Furthermore, the GeneHancer enhancers and their connections are rated as either “elite” or “nonelite” based on reaching a threshold of supporting evidence from multiple different experimental assays or computational methods. As before, we used Poisson GLMs with a log link function; in each model, unless otherwise specified, we controlled for the effects of allele length (as an offset variable), repeat unit length, and GC fraction (both as covariates). STRs in GeneHancer enhancers have significantly fewer interruptions than those outside (Poisson GLM, *B* = −0.035, *P* < 2 × 10^−16^). However, STRs in GeneHancer enhancers are also significantly less likely to be pure (binomial GLM, controlling for allele length, repeat unit length, and GC fraction; *B* = −0.049; *P* = 1.12 × 10^−10^). In essence, STRs in GeneHancer enhancers are more likely to be slightly interrupted, while STRs outside of these elements are either pure or highly impure (Supplementary Fig. 6). This nonmonotonic effect contrasts with our results using chromHMM annotations to infer gene regulatory elements. More in line with the chromHMM results, however, STRs that fall within elite GeneHancer enhancers have significantly fewer interruptions than those falling in nonelite elements (Poisson GLM, *B* = −0.0198; *P* = 4.58 × 10^−5^).

GeneHancer enhancers are annotated with connections to genes whose expression they are inferred to regulate (Fishilevich et al. 2017). We leveraged these annotated gene connections to further stratify the constraint on noncoding STRs. We hypothesized that a regulatory element's constraint could be approximated by the maximum constraint of the genes it regulates. Thus, for all STRs that overlapped elite GeneHancer enhancers, we calculated the minimum LOEUF score of all genes with elite connections to their containing element. Higher maximum genic constraint (i.e. lower minimum LOEUF) is significantly associated with fewer interruptions at STRs that overlap elite GeneHancer enhancers (Poisson GLM, *B* = 0.0269, *P* = 1.27 × 10^−3^). However, containing an elite connection to a known autosomal dominant gene did not significantly affect STR purity (Poisson GLM, *P* = 0.562).

We further examined the effect of constraint on noncoding STR interruptions using a locus-specific score of selective constraint. As described in (Mitra et al. 2021), SISTR is a method that infers the selective cost (*s*) of the gain or loss of a single motif relative to the fitness of an STR locus's major allele. Loci where all possible allele lengths are equally fit experience unconstrained slippage and therefore high variance in allele length (*s* = 0). However, constrained loci at which slippage is deleterious have a lower variance in allele length than would be expected under neutrality (*s* > 0), as estimated by a depletion in heterozygosity observed in a population of 3,200 unrelated individuals (Mitra et al. 2021). We examined 62,729 2-, 3-, and 4-mer noncoding STR loci with reliable *s* scores (Methods); of these, 38,146 were inferred to be under purifying selection. To infer interruptions, we counted the number of nonsingleton substitution single-nucleotide variants (SNVs) identified in gnomAD that fell within the boundaries of each repeat (Chen et al. 2024a). After correcting for covariance with reference allele length (as an offset), GC content, and repeat unit length (both as covariates), we observed that loci under purifying selection harbored significantly fewer interrupting SNVs than those evolving neutrally (Poisson GLM with log link function, *B* = −0.0348, *P* = 4.22 × 10^−10^) (Supplementary Fig. 7). These results appear concordant with our findings using regulatory elements to approximate constraint: higher constraint at noncoding STRs typically predicts higher purity. A caveat for this analysis, however, is that the models of neutral STR variation may not fully account for variance in purity at loci; therefore, the strength and significance of this association may be underestimated. Loci with more cryptic interruptions may harbor lower heterozygosity than expected under SISTR's model, thus appearing under selection. As we observe the opposite association of constraint and interruptions, we are confident in the signal's direction but the true association may be stronger than we infer.

## Discussion

Allele purity is one of many characteristics that determine an STR's instability and mutation rate (Wright et al. 2020; McGinty and Sunyaev 2023; Rajan-Babu et al. 2024 ). At loci under purifying selection, instability can lead to deleterious expanded or contracted mutant alleles; stabilizing a repeat by introducing an interruption is therefore protective against developing those deleterious alleles, as observed at a variety of loci at which expansion causes Mendelian disease (Eichler et al. 1995; Eichler and Nelson 1996; Pearson et al. 1998; Wright et al. 2019, 2020; Rajan-Babu et al. 2024). However, at other loci, purity may itself be under purifying selection; thus, the benefits of an interruption decreasing instability are theoretically counterbalanced by the cost of decreasing purity. At coding STRs, we observed evidence of this tradeoff affecting locus purity: at STRs in highly constrained genes, silent interruptions were favored while missense interruptions were depleted. The opposite was true at STRs in genes evolving more neutrally, at which slippage may be less deleterious and therefore increasing stability less critical. At noncoding STRs, we observe a similar tradeoff occurring. STRs in gene regulatory elements are significantly purer than those falling in putatively neutrally evolving genomic regions: in general, the cost of instability appears less than the cost of impurity. The formation of certain non-B DNA structures seems to modify this effect, particularly at STRs proximal to promoters.

Historically, STRs served largely as neutral markers of ancestry, encoding fine scale demographic information thanks to their high heterozygosity. More recently, however, some STRs are believed to have functional roles in phenotype. Instability at over 60 coding STR loci has been linked to Mendelian diseases; some loci in their larger noncoding counterpart are associated with complex traits and inferred to be under purifying selection (Fotsing et al. 2019; Mitra et al. 2021; Margoliash et al. 2023; Hiatt et al. 2025; Tanudisastro et al. 2025). The results here somewhat support this trend in recognizing that indirect positive selection may act over long periods of time to help shape locus sequence. The effects we report are significant but the effect size is small; though selection may play a role in shaping purity, mutation clearly dominates, as evidenced by the consistent strong associations of purity with allele length, repeat unit, and GC content. Future analyses may be better powered to detect effects of selection after more precisely accounting for mutation rate variation. These models could build in predicted non-B DNA structures, nonlinear effects of certain motifs, or myriad other local determinants of the instability that leads to interruptions. For example, several recent studies predict nonlinear effects of allele length on instability, which theoretically implies a length-dependence in the selective benefit of an interruption (Ananda et al. 2013; McGinty et al. 2025). Even more complex models could account for both the gain and loss of interruptions through slippage (McGinty and Sunyaev 2023). Furthermore, we chose proxies of STR constraint that we believed were unlikely to affect the neutral mutational processes that create new interruptions. These assumptions of independence could be incorrect. For example, if expansions generally lead to higher rates of new interruptions at STR loci in heterochromatin than in euchromatin, our interpretation of constraint explaining an observation of greater purity at loci in gene regulatory elements would be compromised. However, at minimum, the results could point to novel annotations that predict neutral mutational processes.

The weak effect of selection on purity could also result from the small absolute difference in selection coefficients between an interrupted and a pure locus. Lower long-term effective population size may also partially explain the small effect of selection on purity. If this difference in selection coefficient is under (2 × *N_e_*)^−1^, the alleles will drift under near-neutrality (Ohta 1973, 1992 ). Although we do not expect demography to affect the directionality of our results, the effect will be stronger in populations or species of larger effective population sizes. Future work could test this by extending similar analyses to compare the purity of observed major STR alleles between species with contrasting demographic history, such as wild and laboratory mice. Furthermore, interruptions at very long STR loci could be more relatively beneficial due to their inherent high instability (Ananda et al. 2013). Furthermore, covariance with recombination rate could further obscure the efficacy of selection by lowering the effective population size through linkage to nearby constrained elements. This phenomenon, dubbed “background selection”, is pervasive throughout the human genome (McVicker et al. 2009; Pouyet et al. 2018). However, recombination may also affect the rate of expansions, contractions, and new interruptions at STRs, although those effects have not yet been estimated genome-wide. Future work could expand the model architecture to account for the possibly nonlinear effects of recombination rate on purity. Finally, this current study is limited to loci spannable by short read next-generation sequencing technologies; a future study may integrate or analyze separately the purity of long STRs.

Our analyses on the observed major STR alleles rely on a highly simplistic assumption that ancestral versions of the alleles were pure, which is unlikely (McGinty and Sunyaev 2023). However, our results hold as long as selective constraint or function does not affect the probability of gaining an interruption, which itself is a neutral mutational process. A future analysis could gain accuracy by instead counting interruptions relative to alleles at homologous loci inferred at each most recent common ancestor of humans and other great ape species, which could be possible with new long read genome assemblies (Kronenberg et al. 2018; Yoo et al. 2025). Nevertheless, our analyses of segregating SNVs that interrupt an STR do not rely on such assumptions of purity and instead polarize to the reference allele, which is a decent approximation of a recent ancestral allele. These results demonstrate that constrained noncoding STRs remain depleted for interruptions even in more recent evolutionary timescales.

The large number of local determinants of instability at STRs can serve as a feature for studying their mutation rate variation: the parameter space is very large. As we observed, selection against instability tunes purity, which is just one easily alterable sequence variable that affects mutation rate. The flexible framework developed here could be easily used to test the dynamics of selection on any other alterable *cis*-acting mutation rate modifier, such as the complexity of the flanks or proximity to other STRs. Furthermore, high quality genotypes are currently being generated for the longer and more complex variable number tandem repeats (VNTRs); the framework is similarly flexible to examine evolution of stability at these loci as altered by their unique characteristics (Nurk et al. 2022; Liao et al. 2023; Danzi et al. 2025; Yoo et al. 2025).

Supplemental material available at GENETICS online.

## Supplementary Material

iyag080_Supplementary_Data
