# Supplementary material for: The selective dynamics of interruptions at short tandem repeats
Source: Genetics. 2026 Mar 25;233(1):iyag080. doi: 10.1093/genetics/iyag080 (PMC13147528; doi:10.1093/genetics/iyag080)
Supplement: iyag080_Supplementary_Data [file iyag080_supplementary_data.zip › Supplemental_Figure_1_GENETICS-2026-309027.docx]

**Supplemental Figure 1**: Schema for determining the number of interruptions at coding STRs at a hypothetical polyglutamine repeat. At each locus, we selected the major allele and determined the number of substitutions between the observed allele and a pure repeat of the same length. We classified each codon as being pure or interrupted. Silently interrupted codons only contained synonymous substitutions, while any missense substitution classified a codon as missense. Alleles with nonsense substitutions were excluded from the dataset.
