# Supplementary material for: The selective dynamics of interruptions at short tandem repeats
Source: Genetics. 2026 Mar 25;233(1):iyag080. doi: 10.1093/genetics/iyag080 (PMC13147528; doi:10.1093/genetics/iyag080)
Supplement: iyag080_Supplementary_Data [file iyag080_supplementary_data.zip › Supplemental_Figure_4_GENETICS-2026-309027.docx]

**Supplemental Figure 4:** interruptions at STRs in chromHMM enhancers proximal to DNA binding protein binding sites. Asterisks indicate that AG dinucleotide repeats were significantly more interrupted than AC repeats (Bonferroni *P* < 0.05).
