# Supplementary material for: The selective dynamics of interruptions at short tandem repeats
Source: Genetics. 2026 Mar 25;233(1):iyag080. doi: 10.1093/genetics/iyag080 (PMC13147528; doi:10.1093/genetics/iyag080)
Supplement: iyag080_Supplementary_Data [file iyag080_supplementary_data.zip › Supplemental_Figure_5_GENETICS-2026-309027.docx]

**Supplemental Figure 5**: motifs predicted to form certain non-B DNA structures vary in their frequency among noncoding STR loci. For all noncoding non-homopolymer STR loci, we ran nBMST to predict whether six possible non-B DNA structures could form within their observed major alleles. APR: A-phased repeats, which form bent DNA; DR: direct repeats, which form slipped strands; GQ: G-quadruplex; IR: inverted repeats, which can form cruciforms; MR: mirrored repeats, which can form triple helix; Z: Z-DNA.
