# Supplementary material for: The selective dynamics of interruptions at short tandem repeats
Source: Genetics. 2026 Mar 25;233(1):iyag080. doi: 10.1093/genetics/iyag080 (PMC13147528; doi:10.1093/genetics/iyag080)
Supplement: iyag080_Supplementary_Data [file iyag080_supplementary_data.zip › Supplemental_Figure_7_GENETICS-2026-309027.docx]

**Supplemental Figure 7**: noncoding STRs under purifying selection have fewer interrupting SNVs than those evolving neutrally.
